# *Arabidopsis thaliana* FANCONI ANAEMIA I (FANCI) has roles in the repair of interstrand crosslinks and CRISPR‐Cas9 induced DNA double strand breaks

**DOI:** 10.1111/tpj.70533

**Published:** 2025-10-22

**Authors:** Atheer Balobaid, Wanda M. Waterworth, Sophya F. Vila Nova, Barry Causier, Vinay Sharma, Madeline R. Park, Manish K. Pandey, Christopher E. West

**Affiliations:** ^1^ Faculty of Biological Sciences University of Leeds Leeds LS2 9JT UK; ^2^ Center of Excellence in Genomics & Systems Biology (CEGSB) Hyderabad India; ^3^ Center for Pre‐Breeding Research (CPBR) International Crops Research Institute for the Semi‐Arid Tropics (ICRISAT) Hyderabad India

**Keywords:** Fanconi anaemia, NHEJ, DNA repair, interstrand crosslinks

## Abstract

DNA repair is crucial for genome stability, in particular for plants which are exposed to high levels of damage arising from UV irradiation, soil pollutants and reactive oxygen species. Damage that affects both strands of the DNA duplex is harder to repair due to both the lack of a template strand and the potential for physical separation of fragmented chromosomes. As such, DNA double‐strand breaks (DSBs) and interstrand DNA crosslinks (ICL) are particularly cytotoxic forms of damage. Here we report the functions of FANCONI ANAEMIA I (FANCI), an *Arabidopsis thaliana* homologue of the mammalian ICL repair protein. We show that in plant cells, as in mammals, FANCI forms a nuclear localised complex with FANCD2. Genetic analysis of plants lacking FANCI displays significant hypersensitivity to the DNA crosslinking reagent mitomycin C. Furthermore, mutation of *FANCI* in combination with mutations in a second ICL repair factor, *METHYL METHANESULFONATE AND UV‐SENSITIVE PROTEIN 81* (*MUS81*), results in increased levels of programmed cell death compared to the corresponding single mutants, revealing roles in maintaining plant genome stability. Sequence analysis of mutational repair of CRISPR‐Cas9‐induced DSBs revealed that FANCI promotes single nucleotide insertions and reduces longer deletions. This pattern of mutations may reflect roles for FA proteins in replication‐coupled repair of a subset of DSBs. Taken together, this analysis finds evidence for multiple roles for FANCI in the maintenance of plant genome stability.

## Significance Statement

DNA repair is essential for plant growth and development. However, the pathways for interstrand DNA crosslink (ICL) repair are not fully characterised in plants. Here we report that a factor important for ICL repair in mammals has similar functions in plants but finds additional roles influencing the repair of DNA double strand breaks. These findings advance our understanding of plant DNA damage responses, important for crop yields and plant survival under changing climates.

## INTRODUCTION

Maintenance of genome integrity is crucial for plant growth and development in addition to safeguarding the fidelity of genetic information. However, due to their sessile, autotrophic lifestyle, plant cells are continuously exposed to both environmental factors which cause genome damage, such as UV and soil contaminants in addition to endogenous by‐products of metabolism, in particular reactive oxygen species (Britt, 1995; Waterworth et al., 2011). Additionally, DNA damage and DNA replication stress arise from both errors in transcription and blocks to DNA polymerase. A wide range of damage products arises in genomic DNA as a consequence of these endogenous and environmental stresses. Unrepaired DNA damage results in the formation of mutations in the plant genome, resulting in compromised plant growth and potentially leading to cell death. Consequently, plants, as other eukaryotes, have multiple DNA repair pathways and DNA damage response mechanisms to combat the constant threat to genome integrity (Hu et al., 2016). Forms of DNA damage that affect both strands of the duplex are particularly cytotoxic and mutagenic; these include DNA double‐strand breaks (DSBs) and interstrand DNA crosslinks (ICLs). DSBs, representing chromosomal breaks, are repaired by either homologous recombination (HR) or random joining of broken DNA ends by non‐homologous end joining (NHEJ) or DNA polymerase theta‐mediated end joining (TMEJ) (Kamoen et al., 2024). End‐joining activities predominate in the G1 phase of the cell cycle, whereas HR is important during DNA replication and in G2 (Hustedt & Durocher, 2016). ICLs can be caused by endogenous factors such as formaldehyde that physically crosslink the two strands of the DNA duplex, preventing transcription and DNA replication. In addition, some plants produce psoralens that result in DNA crosslinks when activated by UVA (Semlow & Walter, 2021).

At a blocked replication fork, the stalled DNA polymerase complex stimulates DNA repair mediated by the Fanconi Anaemia (FA) pathway. The basic model of ICL repair involves detection of a stalled replication fork by the FANCM complex followed by recruitment of a ubiquitin ligase complex. This ubiquitinates a dimer of FANCD2 and FANCI (the DI complex) which results in recruitment of repair factors. The ICL is ‘unhooked’ by endonucleases to generate a DNA end and a monoadduct which can be bypassed by a translesion polymerase and subsequently removed by nucleotide excision repair (Semlow & Walter, 2021). Homologous recombination then restores the replication fork. In mammals, this ICL repair pathway is mediated by four groups of protein complexes (Semlow & Walter, 2021). The first consists of FANCM with three accessory proteins which together are responsible for detecting blocked replication forks. This complex is largely conserved in plants, although it has only minor roles in interstrand crosslink repair when compared to the central importance of the FA pathway in mammals (Crismani et al., 2012; Dangel et al., 2014; Girard et al., 2014; Knoll et al., 2012). After ICL detection, an E3 ubiquitin ligase complex is recruited, consisting of nine proteins, three of which have homologues identified in Arabidopsis (Semlow & Walter, 2021; Singh et al., 2023). This complex ubiquitinates both FANCD2 and FANCI of the DI complex and is required for ICL repair in mammals (Smogorzewska et al., 2007). Ubiquitination in response to DNA damage requires the activity of the DNA replication stress signalling kinase ATR (ATM AND RAD3 RELATED) (Andreassen et al., 2004). Finally, multiple pathways mediate unhooking, lesion bypass and reconstruction of the replication fork, including nucleotide excision repair, translesion DNA polymerase and homologous recombination (HR) (Semlow & Walter, 2021). These DNA repair factors are largely conserved in plants, including the ICL repair helicase FANCJ (Dorn et al., 2019) and the downstream repair pathways (Bray & West, 2005). Homologous recombination proteins involved in ICL include RAD51 (also named as the FA factor FANCR), BRCA1 (FANCS) and BRCA2 (FANCD1), with functions downstream of ICL detection to re‐establish the replication fork (Michl, Zimmer, & Tarsounas, 2016). BRCA1 and BRCA2 also play important roles earlier in response to DNA replication stress, stabilising DNA replication forks that have stalled at DNA polymerase blocks including R‐loops (RNA stably annealed to the duplex), G quadruplexes (secondary structures formed a G‐repeats) and DNA protein complexes (Michl, Zimmer, & Tarsounas, 2016).

In plants, ICL repair occurs by parallel mechanisms with less reliance on the main FA pathway than in mammals. However, many homologues of FA proteins are conserved including FANCD2 and FANCI (Enderle et al., 2019). To date, the study of plant FANCI and FANCD2 has been largely confined to meiosis. During the early stages of meiosis, programmed DSBs are repaired by HR, resulting in alignment and crossovers (CO) between homologous chromosomes, facilitating the correct segregation of chromatids into gametes. CO frequency is determined by the activity of pathways that promote and resolve recombination intermediates, including components of the ICL repair pathway (Seguela‐Arnaud et al., 2015). In Arabidopsis, FANCD2 was shown to stimulate COs, with *fancd2* mutants displaying reduced meiotic recombination (Kurzbauer et al., 2018). Similarly, in a screen for mutations that increase CO frequencies to restore fertility in a CO mutant, *fanci* and *fancd2* knockouts failed to rescue the mutant; in contrast, *fancm* lines significantly increased seed production in CO mutants (Girard et al., 2014). Thus, FANCM is an anti‐CO factor in plants, whereas the DI complex enhances CO frequencies (Crismani et al., 2012; Girard et al., 2014). This requirement for FANCI and FANCD2 in efficient homologue pairing is reflected in the increased incidence of univalents (unpaired chromosomes) in *fanci* and *fancd2* mutants (Girard et al., 2014; Kurzbauer et al., 2018). However, the functions of the DI complex in ICL repair in plants are less well characterised.

Here, we establish roles for FANCI in DNA repair in Arabidopsis. We show that FANCI forms a protein complex with FANCD2 in plant cells, consistent with the conservation of a functional DI complex in plants. Analysis of FANCI deficient mutants identifies significant hypersensitivity to the ICL reagent mitomycin C. Furthermore, double mutants deficient in both FANCI and MUS81 exhibit increased levels of programmed cell death compared to the corresponding single mutants. Analysis of repair of CRISPR‐Cas9 induced breaks in different sequence contexts reveals unexpected roles for FANCI in DSB repair. These results were further supported by analysis of DNA repair products in FANCD2 and BRCA1 deficient lines. Taken together, these results identify important roles for FANCI in the maintenance of plant genome stability.

## RESULTS

### Arabidopsis FANCI sequence analysis

**Figure 1:**
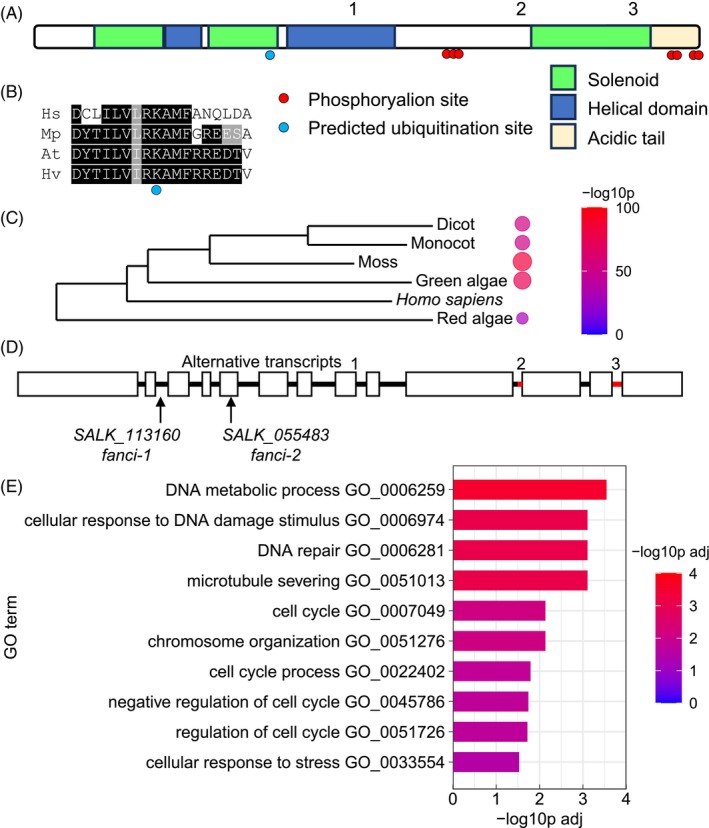
Sequence analysis and expression of *FANCI* in Arabidopsis. (A) Primary structure of FANCI showing the solenoid domains (Pfam 14 675, 14676 and 14678) and helical domains (Pfam 14 679 and 146 800). Phosphosites identified in published studies are indicated by red circles, and a conserved lysine that is the site for ubiquitination in mammals is indicated by a blue circle. (A) C‐terminal 111 aa acid domain has 35% aspartate and glutamate residues. The positions of sequence differences between alternative transcripts from part (D) are indicated by the numbers. (B) Alignment of the ubiquitinylated domain in FANCI proteins from plants and mammals. Hs: *Homo sapiens* FANCI AAI40770.1; Mp: *Marchantia polymorpha* putative FANCI PTQ38472.1; AT: *Arabidopsis thaliana* FANCI AT5G49110.3; Hv: *Hordeum vulgare* putative FANCI XP_044981537.1. (C) Phylogenetic tree of the aligned full‐length FANCI sequences from part (B) together with representatives from green algae (GAB4818334.1) and red algae (PXF46357.1). Tree generated at https://www.phylogeny.fr/. BLAST *P* values from pairwise alignments of plant FANCI with the human orthologue are shown. (D) Genomic organisation of FANCI showing the positions of the T‐DNA insertion lines *fanci‐1* and *fanci‐2* used in this study. Alternative splice sites are indicated as in part (A), and the additional intron sequence in *AT5G49110.3* (relative to *AT5G49110.1*) is indicated in red. With reference to *AT5G49110.1*, *AT5G49110.3* has an additional glutamine at the splice site at the end of exon 8 (1); an additional 12 aa at the start of exon 11 (2); and 24 additional amino acids by inclusion of intron 12 (3). (E) Gene ontology enrichment of genes co‐expressed with *FANCI*. Co‐expressed genes are identified, and gene ontology enrichment was analysed using www.michalopoulos.net/act/.

Sequence analysis of FANCI has been previously reported (Girard et al., 2014). The *FANCI* gene (*AT5G49110.1* and *NM_001344814*) consists of 13 exons encoding a 1431aa protein containing solenoid and helical motifs as identified in human FANCI (AAI40770.1) (Figure 1). Multiple phosphorylation sites include three around residue 860 and multiple sites in the C‐terminal region, with S1415 identified in several tissue types in published proteomics data (Mergner et al., 2020; athena.proteomics.wzw.tum.de). Lysine 520 corresponds to the site of ubiquitin‐mediated regulation in mammals and is conserved among plant and animal FANCI proteins (Figure 1B). BLAST analysis revealed significant similarity between human FANCI and putative orthologues in plants and algae (p values ranging from 10^−25^ to 10^−99^; Figure 1C) (Girard et al., 2014). Predicted alternative *FANCI* transcripts displayed differences in splicing at the boundaries of exon 8/intron 8 and intron 9/exon 10, in addition to variable retention of intron 12 (Figure 1D; positions 1, 2 and 3). The splice variant identified in this study corresponded to TAIR sequence *AT5G49110.3* which differs from *AT5G49110.1* in using two alternative splice sites within introns and retaining intron 12, coding for additional 1, 12 and 24 amino acids, respectively. The positions of the *fanci‐1* and *fanci‐2* mutants are indicated (Figure 1D), and both mutant lines disrupt *FANCI* expression (Figure S1). *FANCI* is co‐expressed with genes enriched in ontology functions including DNA repair and cell cycle (Figure 1E) and was previously identified as being responsive to gamma radiation (Culligan et al., 2006).

### FANCI interacts with FANCD2

**Figure 2:**
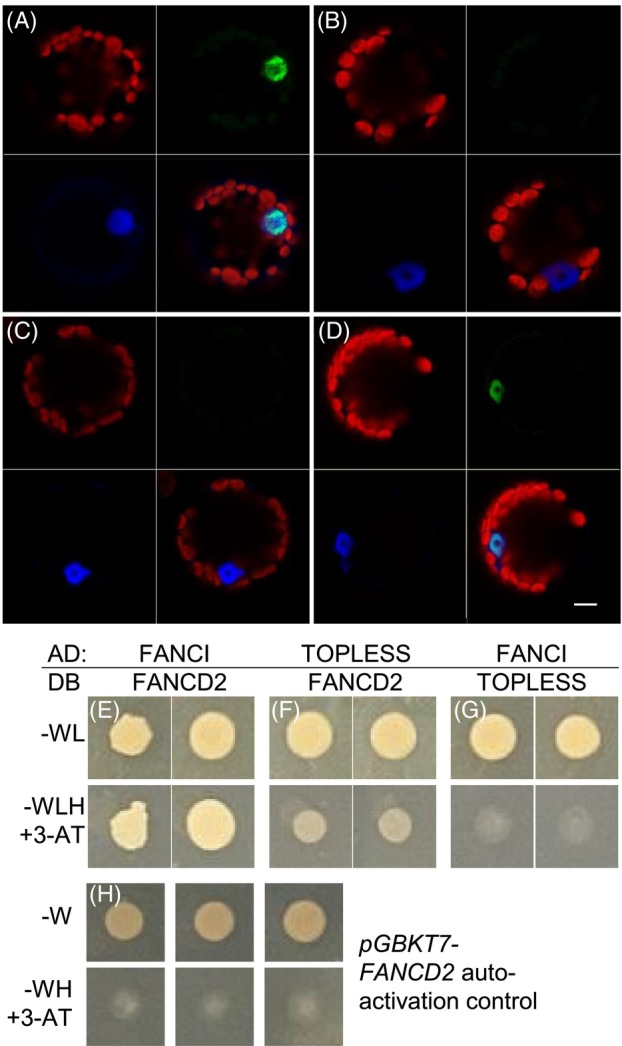
Interaction between FANCI and FANCD2. (A–D) Bimolecular fluorescence complementation. Laser scanning confocal microscopy of Arabidopsis protoplasts transformed with cYFP and nYFP protein fusion expression constructs. Protoplasts were imaged 24‐h post‐transformation using filters for chlorophyll, dsRED and YFP. Chlorophyll autofluorescence is shown in red, dsRED‐nls in blue and YFP in green. (A) MRE11‐nYFP + HAF1‐cYFP‐positive control for protein interaction. Negative controls consisted of either FANCI or FANCD2 in combination with ACTIN2: (B) ACTIN2nYFP + FANCI‐cYFP. (C) FANCI‐nYFP + ACTIN2cYFP (D) FANCI‐nYFP + FANCD2‐cYFP test interaction. Scale bar is 10 μm. (E–G) Confirmation of FANCI‐FANCD2 interaction by yeast two hybrid. (E) Interaction between FANCI‐GAL4 activation domain (AD) and FANCD2‐GAL4 DNA‐binding domain (DB) results in growth on histidine‐deficient media supplemented with 3‐AT (3‐Amino‐1,2,4‐triazole). Media lacking tryptophan and leucine (‐WL) show growth of the transformed yeast whereas media additionally lacking histidine (‐WLH) only allows growth in the presence of interacting bait and prey proteins. (F–G) Low growth on selective media for negative control interactions with TOPLESS (AT1G15750). (H) Low levels of autoactivation in yeast expressing FANCD2‐DB are shown by poor growth on histidine‐deficient media.

Human FANCI was shown to interact with FANCD2, both *in vitro* and HeLa cells, forming the DI complex (Yuan et al., 2009). To test the interaction between Arabidopsis FANCI and FANCD2, plasmids expressing full‐length proteins fused with the N‐terminus of YFP (nYFP) and the C‐terminus of YFP (cYFP), respectively, were transfected into Arabidopsis protoplasts to test for bimolecular fluorescence complementation (Zhong et al., 2008). As a positive control, MRE11‐nYFP was co‐transfected with HAF1c‐YFP. Reconstitution of YFP resulted in nuclear localised fluorescence indicative of interaction between MRE11 and HAF1, as previously reported (Figure 2A) (Waterworth et al., 2015). Similarly, YFP fluorescence in protoplasts transformed with FANCD2‐nYFP and FANCI‐cYFP demonstrated interaction between these proteins and formation of the DI complex in the nucleus of plants (Figure 2D), whereas YFP fluorescence was absent from controls (Figure 2B,C). This demonstrated the formation of a plant FANCD2 FANCI (DI) complex as previously reported for mammalian cells (Alcon et al., 2020). Confirmation of this interaction was provided using a yeast two hybrid. In a negative control, fusion of FANCD2 to the GAL4 DNA binding domain (DB) resulted in low levels of autoactivation in yeast reporter lines (Figure 2H). However, the addition of the FANCI‐GAL4 activation domain (AD) fusion led to histidine prototrophy (Figure 2E), indicative of FANCI‐FANCD2 interaction. In contrast, controls where FANCI or FANCD2 was replaced by the transcription factor TOPLESS (AT1G15750) failed to rescue histidine auxotrophy (Figure 2F,G).

### Mutant *fanci* plants are hypersensitive to mitomycin C

**Figure 3:**
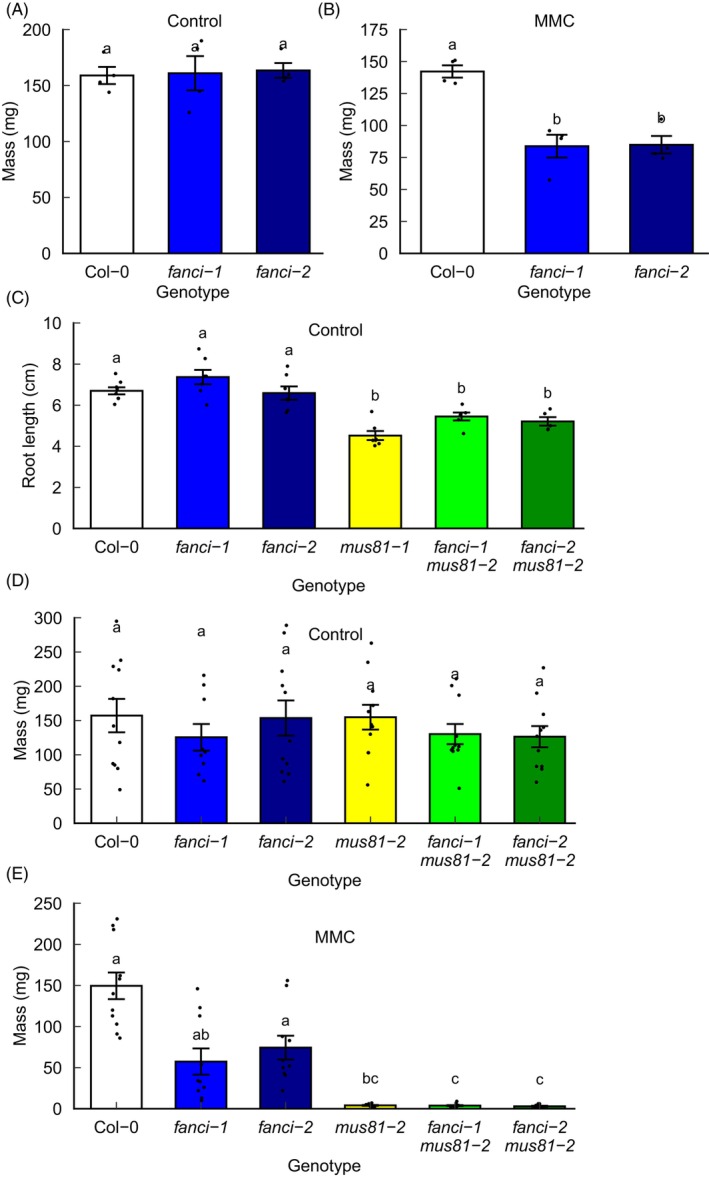
Reduced growth of *fanci* and *mus81* single and double mutants in the presence of mitomycin C. (A, B) Mean plant mass from pooled data from >4 plants per treatment (*n* = 4). (A) Control treatment after 3‐week growth (B) 5‐week growth after exposure to 6 mg/L MMC. Different letters denote significantly different groups (*P* < 0.01, ANOVA with Tukey correction). (C) Root length of *fanci* and *mus81* single and double mutants grown for 3 weeks on vertical ½ MS agar plates. Different letters denote significantly different groups (*P* < 0.01, ANOVA with Tukey correction, *n* = 5–8). (D) Average seedling mass after 3‐week growth in control media or (E) 5‐week growth in media supplemented with 6 mg/L MMC. Data represent means ± SE. Different letters denote significantly different groups (*P* < 0.05, Kruskal–Wallis with Bonferroni correction; *n* = 10–11). Data points represent mean values of >10 plants (A, B) or individual plants (C–E).

No difference was observed in the growth and development of *fanci* mutant lines and wild‐type controls in terms of shoot and root growth (Figure S2), suggesting that the absence of FANCI does not affect plant development under optimal growth conditions. To determine the role of FANCI in DNA repair in Arabidopsis, wild‐type and mutant lines were exposed to a range of genotoxins and growth was compared to controls. Mutants lacking FANCI displayed wild‐type levels of sensitivity to UVC, X‐rays and hydroxyurea, suggesting that the FA pathway is not essential for survival under conditions of elevated single or double‐stranded DNA damage or replication stress (Figure S3). To assess the role of FANCI in plant resistance to interstrand DNA crosslinks (ICLs), sensitivity to the bifunctional alkylating agent mitomycin C (MMC) was assessed. Previous analysis of Arabidopsis *fancm* mutants reported wild‐type levels of MMC sensitivity (Crismani et al., 2012; Knoll et al., 2012). In contrast, the repair exonuclease MUS81 was shown to be involved in ICL repair with *mus81* mutants displaying high MMC sensitivity (Hartung et al., 2006). Crossing mutant plants defective in ICL repair can be used to determine whether different repair factors act within the same pathway or in parallel pathways of repair (Enderle et al., 2019). Plants mutated in both MUS81 and FANCM displayed very poor growth, dying shortly after germination in one study (Dangel et al., 2014) and resulting in weak but viable plants in a separate study using different mutant alleles (Crismani et al., 2012). This requirement for FANCM in MUS81‐deficient plants suggested roles for FANCM in ICL repair in parallel to MUS81 (Crismani et al., 2012; Dangel et al., 2014). Here, the role of FANCI in the repair of interstrand crosslinks was studied by comparing the effect of MMC on the fresh weight of Col‐0 and *fanci* single mutant plants and in combination with *mus81* mutation. Exposure to MMC revealed mild but significant hypersensitivity of *fanci* mutants (Figure 3B; Table S1). In contrast to *fancm mus81* mutants, *fanci mus81* plants developed normally. In the absence of treatment, root length was significantly reduced in single and double mutants lacking MUS81 (Figure 3C), but no significant difference in fresh weight was observed between wild‐type and mutant lines (Figure 3D). Single and double mutant lines lacking MUS81 displayed severe MMC hypersensitivity relative to the mild phenotype of *fanci* lines (Figure 3E), consistent with important roles for MUS81 in ICL repair as demonstrated in previous studies (Hartung et al., 2006). These results support a weak role for FANCI in ICL repair, consistent with published plant FA mutants and in contrast to mammalian FA‐deficient lines (Crismani et al., 2012; Dangel et al., 2014; Enderle et al., 2019; Knoll et al., 2012).

### Elevated cell death in *fanci* mutants

**Figure 4:**
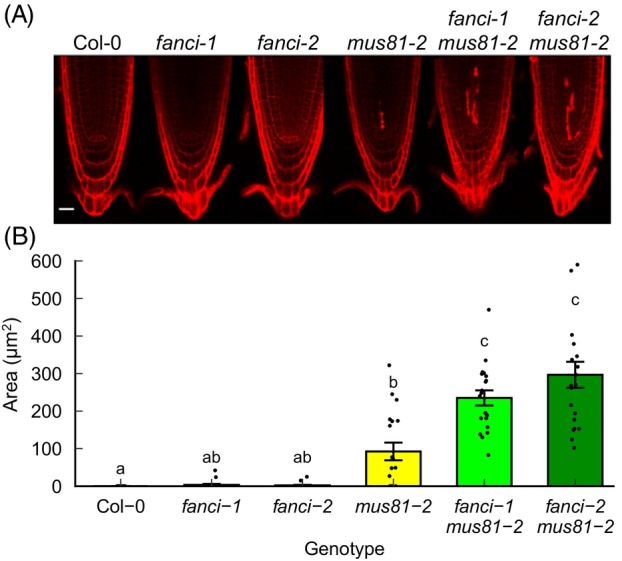
Programmed cell death in the root apical meristem of *fanci* and wild type plants. (A) Representative images of confocal planes from the PI‐stained root apical meristem of wild type Col‐0 plants and *fanci* and *mus81* single and double mutants. (B) Quantification of cell death. Data represent means ± SE and letters represent homogeneous groups (Kruskal–Wallis test with Bonferroni correction, *P* < 0.05, *n* = 18–21 roots). Data points represent total area of dead cells per root.

Plants deficient in DNA repair, including mutant lines lacking Fanconi anaemia homologues, have been shown to display constitutively elevated levels of cell death in the root apical meristem (RAM) (Dorn et al., 2019; Fulcher & Sablowski, 2009). Here, single and double mutants lacking FANCI and MUS81 were tested for cell death in the RAM under standard growth conditions. Low levels of cell death were observed in Col‐0 and *fanci* mutants, as revealed by propidium iodide viability staining and fluorescence microscopy (Figure 4; Figure S4; Table S2). In contrast, the majority of roots of *mus81* mutants and all roots of *mus81 fanci* double mutants displayed cell death. Quantification of cell death area in the optical section containing the quiescent centre (QC) cells revealed significantly greater levels of cell death in *mus81* mutants relative to Col‐0. Furthermore, *mus81 fanci* double mutants displayed greater cell death than the corresponding single mutants. These results are consistent with non‐redundant roles for FANCI and MUS81 in protecting dividing cells from genome stress.

### FANCI deficient plants display altered DSB repair profiles

**Figure 5:**
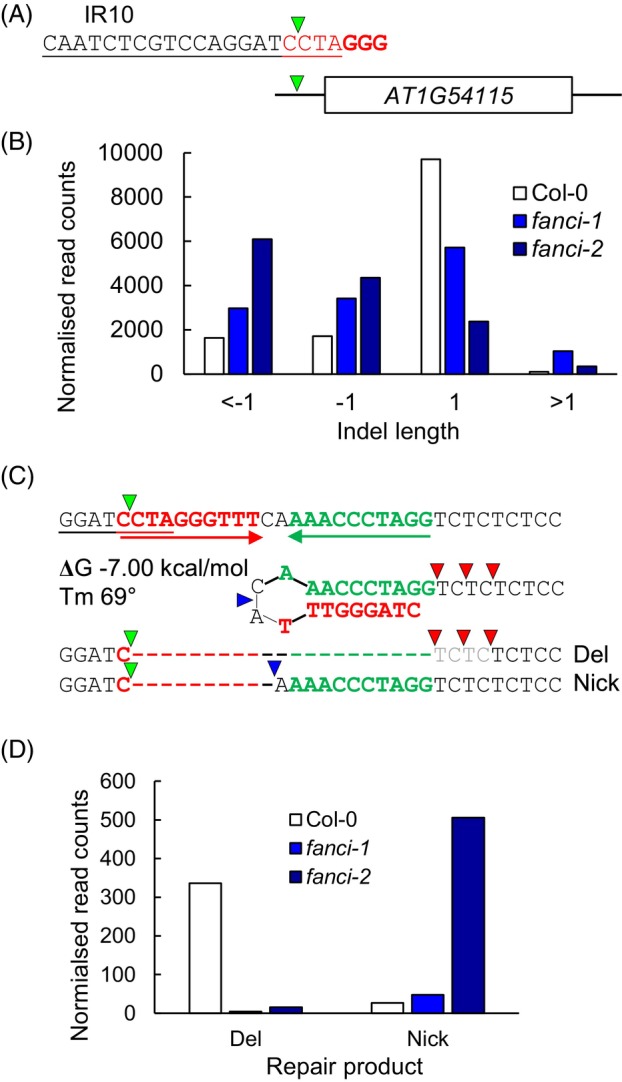
Characterisation of repair at a DSB induced next to an inverted repeat in wild type and *fanci* mutants. (A) The protospacer sequence at the IR10 inverted repeat with the PAM sequence shown in bold. (B) Analysis of amplicon sequencing products quantifies the numbers of insertions and deletions of 1 bp or more. The mutant lines display significantly different distributions of repair products (*P* < 0.001, chi squared). Mapped reads: Col‐065430, 19% modified, *fanci‐1*50 667, 38% modified, *fanci‐2*47 611, 8% modified. (C) Predicted folding of the repeats, indicated in green and red, produces a hairpin structure with a calculated Tm of 69°C (www.unafold.org/). Resolution by nicking produces a 10 bp deletion whereas removal of the repeat and microhomology mediated repair (indicated by red triangles) leads to a 25 bp deletion. Cas9 occasionally cuts at the −4 position from the PAM, instead of the −3 (de Pater et al., 2024), which would create a 1 bp longer hairpin. (D) Quantification of the hairpin resolution deletion products in wild type and *fanci* mutant plants.

To further analyse the role of FANCI in DNA repair, amplicon sequencing was used to characterise repair of DSBs in wild type and *fanci* mutant lines. Inverted repeats have the potential to cause DNA replication stress as single‐stranded DNA at replication forks could self‐anneal. To investigate the roles of FANCI in DSB repair, a target site was identified within the 5′UTR of *CATION CALCIUM EXCHANGER 4* (*AT1G54115*) that contained a 10 bp inverted repeat (IR10) separated by a 2 bp spacer (Figure 5). CRISPR‐Cas9 was used to induce a DSB at the start of the inverted repeat. PCR amplification and deep sequencing of the amplicons was followed by analysis using CRISPResso (Pinello et al., 2016). As previously reported, the majority of mutations at a Cas9‐induced break in Arabidopsis are single nucleotide insertions (de Pater et al., 2024). However, *fanci* mutants displayed a significant reduction in the frequency of single nucleotide insertions relative to wild type, with a corresponding increase in larger deletions (*P* < 0.001, Chi squared test; Figure 5B). A small fraction of reads displayed products consistent with resolution of inverted repeat hairpins, either through nicking of the hairpin loop or removal of the hairpin (Figure 5C). As these products accounted for less than 1% of reads, they could represent single repair events which precludes comparisons of frequencies between samples. Thus, these data does not provide evidence of any significant difference between wild type and mutant alleles in the resolution of hairpin structures. However, the spectrum of products is consistent with the presence of hairpin nicking activity in both wild type and *fanci* mutant lines (Figure 5D).

**Figure 6:**
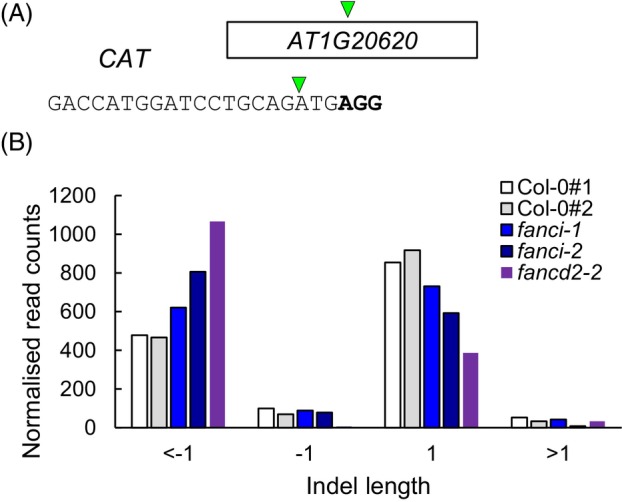
Mutation characterisation of wild type and *fanci* mutants at a DSB induced in the *CAT3* gene. (A) The protospacer sequence designed for *CAT3* with the PAM sequence shown in bold. (B) Analysis of amplicon sequencing products quantifies the numbers of insertions and deletions of 1 bp or more. The *fanci‐1*, *fanci‐2* and *fancd2‐2* mutant lines display significantly different distributions of repair products in comparison to wild type (*P* < 0.001, chi squared). Mapped reads: Col‐0#112771, 11% modified, Col‐0#2 6799, 8% modified, *fanci‐1* 37590, 4% modified, *fanci‐2* 9407, 7% modified, *fancd2‐2* 53769, 16% modified.

The difference in end‐joining activity in *fanci* mutants was unexpected; a different CRISPR‐Cas9 construct with a target site within *CATALASE3* (*CAT3*, *AT1G20620*) was tested to determine whether the results at the IR10 locus were also observed at a locus not containing any repeats. As observed as with the IR10 induced break site, a significant reduction in single nucleotide insertions and a corresponding increase in deletions longer than 1 nucleotide were found at the induced break in *CAT* in *fanci* mutant lines relative to wild‐type controls (Figure 6B). This indicates a role for FANCI in influencing repair outcomes at induced breaks in Arabidopsis. Significantly, a reduction in single nucleotide insertions was also observed in the *fancd2‐2* mutant line. The differences in single insertion frequencies and longer deletions between the *fanci‐1*, *fanci‐2*, and *fancd2‐2* alleles may reflect residual function in some alleles and FANCD2 functions that are independent of the DI complex.

The amplicon sequence data indicated roles for the DI complex in the repair of CRISPR‐Cas9 induced breaks. The FA pathway consists of components for ICL detection and processing, followed by re‐establishment of the replication fork through HR. Thus, HR factors are designated as FA proteins, including BRCA1 (termed FANCS). To investigate the roles of downstream FA components in the repair of induced breaks, amplicon sequence analysis of DSB repair at the IR10 site was investigated in two independent *brca1* mutant alleles, including the previously published allele *brca1‐1* shown to be hypersensitive to MMC (Reidt et al., 2006). In addition to its roles in HR, BRCA1 also helps stabilise replication forks stalled at ICLs and other blocks (e.g. R‐loops), preventing collapse of the replication fork (Michl, Zimmer, & Tarsounas, 2016). The influence of *brca1* mutation on the repair outcomes of Cas9 induced DSBs was investigated using the IR10 target site.

**Figure 7:**
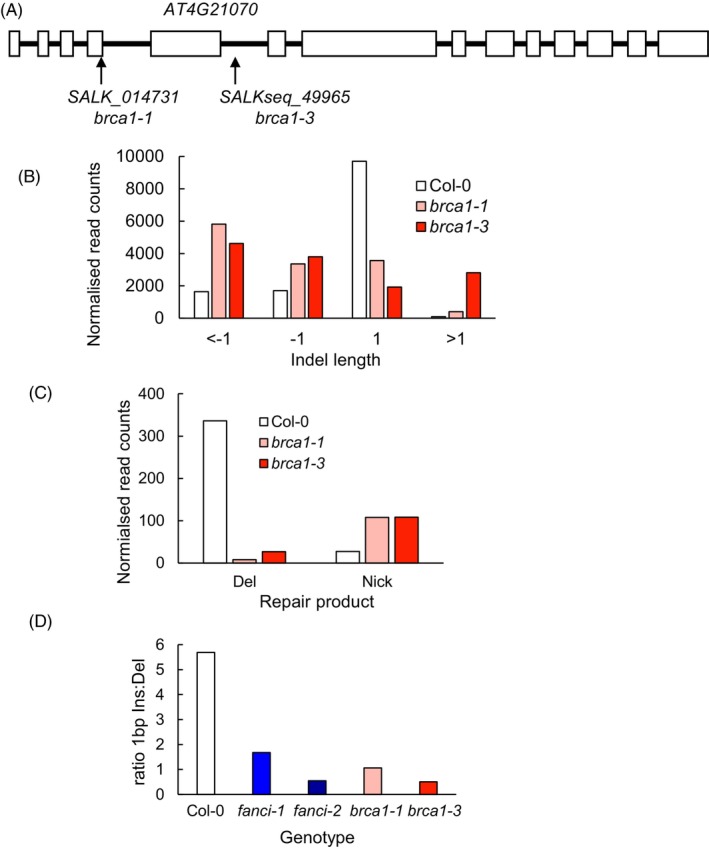
Repair of an induced break at the IR10 locus in *brca1* mutants. (A) Genomic region of *BRCA1* (*AT4G21070*) with exons shown as boxes and introns as lines. The positions of the *brca1‐1* and *brca1‐3* T‐DNA insertion sites are shown. (B) Quantification of insertions and deletions at IR10 as analysed using CRISPResso. Data for Col‐0 are reproduced from Figure 5. (C) Numbers of reads corresponding to putative hairpin repair products arising from nicking (Nick) or removal (Del) as described in Figure 5. (D) Ratio of single nucleotide insertions: single nucleotide deletions in wild type, *fanci* and *brca1* mutant lines based on data presented in Figures 5D and 7C. Mapped reads: Col‐0 (as Figure 5), *brca1‐1*41 987 (68% modified), *brca1‐3*41 577 (72% modified).

As observed for *fanci*, *brca1* mutants displayed a shift from single nucleotide insertions to an increased number of deletions (Figure 7B). When expressed as a ratio of single nucleotide insertions: single nucleotide deletions, wild‐type lines display a ratio of 6 whereas this ratio is between 0.5 and 1.7 in the mutant lines (Figure 7D). Previous reports have highlighted the prevalence of single nucleotide insertions, for example comprising ~90% of mutations at a Cas9 induced break in the *RTEL1* locus in Arabidopsis (Fauser et al., 2014). In a more recent study, mutagenic repair of a Cas9 induced break resulted in 28% single nucleotide insertions at the *PPO* locus and 60% at the *ADH* locus, strongly dependent on the activity of DNA polymerase λ (de Pater et al., 2024). The data presented here provide evidence that activities of FA factors influence the outcome of CRISPR‐Cas9 induced DSB repair in Arabidopsis (Figure 7). This may reflect direct roles for FA proteins in DSB repair or indirect roles in processing of DSBs encountered during DNA replication. Roles for the mammalian FA pathway in promoting HR have been reported, including components of the DI complex (van de Kooij et al., 2024). It is possible that reduced HR in Arabidopsis mutants leads to greater use of alternative repair pathways in cells in S/G2. Analysis of repair products indicates ~2‐fold greater use of microhomology in the *fanci* mutant lines relative to wild type at both the IR10 and CAT locus supportive of the greater use of alternative DSB pathways in the *fanci* lines (Tables S3 and S4. IR10 data excludes products of hairpin removal).

## DISCUSSION

The current study investigates the role of FANCI in Arabidopsis. FANCI forms part of the Fanconi Anaemia (FA) pathway and is primarily known for its role in DNA interstrand crosslink repair in humans, where mutations in FANCI result in genomic instability, bone marrow failure and cancer predisposition (Smogorzewska et al., 2007). ICLs are detected by FANCM together with the accessory proteins MHF1 and MHF2 (Semlow & Walter, 2021), which, in turn, recruit a core complex of proteins and result in ubiquitination and recruitment of FANCD2 and FANCI. Repair factors with nuclease and helicase activities initiate processing of the ICL. Repair is completed by multiple pathways including homologous recombination, nucleotide excision repair, post‐replication repair and translesion synthesis (Semlow & Walter, 2021). Previous work has shown that Arabidopsis has functional homologues of FANCM, MHF1 and MHF2, but that mutants of these factors have minor roles in ICL repair; mutants do not display increased sensitivity to MMC unless combined with *mus81* mutations (Crismani et al., 2012; Dangel et al., 2014; Girard et al., 2014; Knoll et al., 2012). In contrast, the core FA complex is poorly conserved, with only three factors identified in Arabidopsis: FANCC, FANCE and FANCF (Singh et al., 2023). Previously, meiotic roles for FANCD2, FANCI and the core complex were revealed in plants and functions for translesion DNA synthesis in ICL repair have also been characterised (Kobbe et al., 2015), but roles for FANCI in ICL repair were not reported (Girard et al., 2014; Kurzbauer et al., 2018; Singh et al., 2023).

Here using bimolecular fluorescence complementation, we show that in Arabidopsis FANCI associates with FANCD2 *in planta* (Figure 2), forming a complex homologous to the mammalian ‘DI clamp’. Phenotypic analysis revealed that, in contrast to FANCM, *fanci* mutants display significant, although mild, hypersensitivity to the ICL reagent mitomycin C (MMC) (Figure 3). Previous epistasis studies identified three parallel pathways for ICL repair in Arabidopsis, dependent on the nucleases MUS81, FAN1 and RAD1 (Enderle et al., 2019). Downstream of FAN1 are three additional parallel sub‐pathways dependent on RECQ4A, REV3 or RAD5A. These pathways and sub‐pathways are only partially redundant as single mutants (*fan1*, *mus81* and *rad5a*) display MMC hypersensitivity (Enderle et al., 2019; Herrmann et al., 2015). This lack of full redundancy presumably reflects differences in substrate, cell‐cycle stage specificity, a limited repair capacity for each pathway or additional functions of these factors that are common to multiple pathways. The nuclease activity of RAD1, FAN1 and MUS81 is thought to function early in the removal of the ICL (Herrmann et al., 2015). Double mutants in components of these parallel pathways often display lethality or extreme sensitivity to ICLs, which may reflect the build‐up of unrepaired ICLs to toxic levels (Enderle et al., 2019). However, growth defects of *recq4A mus81* or *fancm mus81* mutants could be rescued by knocking out homologous recombination, illustrating that persistence of recombination intermediates, rather than ICLs, resulted in cytotoxicity in these mutant lines (Crismani et al., 2012; Hartung et al., 2006; Mannuss et al., 2010). Here, the *fanci mus81* mutants displayed normal development, as previously observed for the *fancd2 mus81* double mutant (Kurzbauer et al., 2018), but were extremely sensitive to MMC. The relative MMC hypersensitivity of *fanci* and *mus81‐2* mutants indicates that MUS81 mediates the major pathway of ICL repair in plants.

Reduced repair DNA capacity can result in increased levels of spontaneous programmed cell death in the actively dividing cells of the plant root and shoot apical meristems, as first reported in the root apical meristem of Arabidopsis *ku80* and *lig4* NHEJ deficient lines (Fulcher & Sablowski, 2009). However, *fanci* single mutants did not display significantly increased PCD levels compared to wild type lines in the present study (Figure 4). This is comparable to the results of a previous analysis of plants lacking the FA factors FAN1 and FANCJB (Dorn et al., 2019; Herrmann et al., 2015). In contrast, when combined with *mus81*, the absence of FANCI here resulted in significant increases in cell death, providing evidence for roles for FANCI in mitigating genome stress in plants, although in a pathway partially redundant to MUS81. The spontaneous cell death observed in ICL repair mutants may reflect background levels of crosslinks formed by reactive products of cellular metabolism including formaldehyde (Semlow & Walter, 2021). However, FA proteins have wider roles in DNA replication stress beyond ICL repair (Michl, Zimmer, Buffa, et al., 2016; Michl, Zimmer, & Tarsounas, 2016). Replication forks stall at blocks to DNA polymerase progression, and mammalian cells deficient in BRCA1 or BRCA2 are hypersensitive to the accumulation of fork blocking structures including G4 quadruplexes. In the absence of BRCA2, FANCD2 plays essential roles in mitigating the toxic effects of replication stresses and mammalian *fancd2 brca2* double mutants display synthetic lethality (Michl, Zimmer, Buffa, et al., 2016). The fork stabilisation activity is also proposed for the DI complex, suggesting that *fanci* and *fancd2* mutations would increase replication fork collapse and influence the outcome of DSBs repaired during S‐phase. However, our analysis of hydroxyurea sensitivity did not provide evidence to support similar mechanisms in plants (Figure S3).

To further analyse the roles of FANCI in Arabidopsis, repair of CRISPR‐Cas9 induced DSBs was investigated. A target site was identified in the 5´UTR of the *CCX* gene located on chromosome 1. This region contains a 10 bp inverted repeat that could lead to the formation of a hairpin structure following resection of the break or passage of a replication fork. Resolution of the hairpin by nicking is predicted to remove a 10 bp repeat, whereas removal of the hairpin and repair would result in loss of 20–25 bp depending on the use of microhomology. The majority of mutational repair events were typical single nucleotide insertions or short deletions, although some reads were consistent with the formation and removal of the hairpin (Figure 5). Surprisingly, there was a significant reduction in the frequency of single nucleotide insertions in *fanci* mutant lines, consistent with roles for FANCI in influencing the outcomes of DSB end joining. This was unexpected as there is not an established link between the FA pathway and the two major end‐joining pathways of NHEJ or polymerase theta‐mediated end joining (TMEJ). This result was confirmed through analysis of mutational repair at an independent site in the Arabidopsis genome, within the *CAT3* gene in both *fanci* and *fancd2* mutant lines (Figure 6). The role for the plant FA pathway in promoting single nucleotide insertions was further explored through analysis of repair profiles in *brca1* mutants. Arabidopsis BRCA1 was previously characterised as having roles in homologous recombination, displaying sensitivity to MMC but not γ‐irradiation (Reidt et al., 2006). Both *brca* mutant alleles studied here displayed reduced levels of single nucleotide insertion at a Cas9 induced DSB at the IR10 locus, similar to the repair products observed for *fanci* mutants (Figure 7). A role for the FA pathway in DSB repair may arise either through direct involvement of FA components in end joining or, alternatively, the link to DSB repair could be indirect. Previous analysis of mutation products at induced breaks led to a model whereby a fraction of breaks are repaired during DNA replication (de Pater et al., 2024), providing a potential link with the ICL repair machinery. Arabidopsis lines deficient in TMEJ displayed extensive deletions at the sites of induced breaks, with sequence loss often only on one end of a DSB (de Pater et al., 2024). A model was proposed in which a DSB is encountered at converging replication forks to produce four DNA ends, two of which are the site of lagging strand synthesis and prone to deletions, providing an explanation for the observed spectrum of repair products. The interaction between the replication machinery and DSBs would provide an indirect link between the plant FA pathway and repair at the site of Cas9 endonuclease activity, with FA proteins functioning to stabilise the replication machinery at the DSB. In the absence of FA factors, increased levels of replication fork collapse would lead to more frequent deletions with a corresponding reduction in single nucleotide insertions. This feature of the FA pathway in influencing DSB repair outcomes may arise, in part, through repeated cycles of Cas9 cutting, as NHEJ usually produces perfect, non‐mutated repair products at endonuclease induced breaks (Schmidt et al., 2019). However, the dependency on FANCI for single nucleotide insertion is not clear, as these insertions are largely dependent on polymerase λ filling in a staggered Cas9 cut or in non‐templated single nucleotide insertion (de Pater et al., 2024). An alternative explanation for the altered spectrum of repair products in the *fanci* mutant lines comes from the research in mammals, finding roles for FA proteins in promoting HR (van de Kooij et al., 2024). In the absence of *fanci*, increased reliance on TMEJ in S/G2 would lead to greater use of microhomology, as observed at both the CAT and IR10 locus, whereas HR proficient lines would result in perfect repair and counted as unmodified reads in the amplicon assay used here.

In summary, here we show that the FA pathway plays a less crucial role in plants compared to animals which reflects the activity of parallel pathways acting in ICL repair, with MUS81 mediating the major pathway as previously reported (Enderle et al., 2019). However, FANCI plays a significant role in ICL repair and additionally functions to reduce levels of deletions at a DSB. Taken together, these results demonstrate that FANCI is a component of a protein complex that promotes plant genome stability.

## METHODS

### Plant growth

Seed stocks were obtained from the Nottingham Arabidopsis Stock Centre and were in the Columbia (Col‐0) background. FANCI (AT5G49110) mutant lines were identified using T‐DNA Express (https://signal.salk.edu/cgi‐bin/tdnaexpress) and included *fanci‐1* (SALK_113160) (Alonso et al., 2003) and the previously reported *fanci‐2* (SALK_055483C) (Girard et al., 2014). The MUS81 (AT4G30870) mutant line *mus81‐2* (SALK_107515) was described previously (Hartung et al., 2006). The BRCA1 (AT4G21070) mutant line *brca1‐1* (SALK_014731) has been published (Reidt et al., 2006), and *brca1‐3* (SALKseq_49965) was provided by NASC. Seeds of Arabidopsis wild type and mutant lines were surface sterilised by adding a sterilisation solution (0.1% (v/v) Triton X‐100 (Acro, Australia) and 3% (v/v) household bleach) and incubating them for 10 min with occasional gentle mixing by inversion. The solution was then removed, and the seeds were washed three times in sterile dH_2_O. Seeds were grown under sterile tissue culture conditions on half‐strength Murashige & Skoog media with 1% sucrose (Murashige & Skoog, 1962). After 2 weeks of growth, the seedlings were transferred to soil. Plants were grown at a 16 h day at 22°C.

### Plasmids

Plasmids used for CRISPR‐Cas9 induced breaks for amplicon analysis were generated as described previously (Fauser et al., 2014). The IR10 target site was identified using the online inverted repeat finder tool https://tandem.bu.edu/irf/home (Warburton et al., 2004). Protospacer oligos (Table S3) were annealed and ligated into *Bbs*I linearised pEn‐Chimera vector. The pEn‐Chimera containing the *gRNA* expression cassette was then transferred into pDe‐Cas9 vector using LR clonase (Invitrogen) following the manufacturer's recommendations and the resulting binary vector was used for *Agrobacterium*‐mediated plant transformation. Gateway destination vectors pDH51‐GW‐YFPn (N9842, containing N‐terminal 465 bp of YFP) and pDH51‐GW‐YFPc (N9843, containing C‐terminal 252 bp of YFP) for generating FANCI and FANCD2 tagged proteins, respectively, were used as described previously (Zhong et al., 2008). Full‐length *FANCI* coding region (*AT4G14970.3*, 4428 bp) was cloned into pENTR (Invitrogen) and transferred to pDH51‐GW‐YFPc and the FANCI coding region (*AT5G49110.3*, 4287 bp) in pENTR was transferred to pDH51‐GW‐YFPn using gateway cloning with LR clonase. Plasmids were confirmed by nanopore sequencing (Genewiz Plasmid EZ). For yeast two hybrid, genes were cloned in pGBKT7 (DB) and pGADT7‐Rec (AD), transformed into yeast strain AH109 and protein–protein interactions were visualised as yeast growth on selective medium depleted in histidine and supplemented with 2.5 mm 3‐AT as described previously (Causier et al., 2011).

### *Agrobacterium*‐mediated transformation

*Agrobacterium* GV3101 cells were transformed with pDeCas9 using electroporation (MicroPulser Electroporator, Bio‐Rad) followed by selection on LB‐agar plates containing spectinomycin, gentamycin and rifampicin. Floral dip transformation of Arabidopsis plants using *Agrobacterium* was carried out as described previously (Clough & Bent, 1998). Flowering Arabidopsis plants were inverted so that aerial tissues were submerged in *Agrobacterium* suspended in a solution of 5% sucrose and 0.05% Silwet L‐77 (OD_600_ = 1). Dipping was carried out with gentle agitation for 5–10 sec. Subsequently, plants were enclosed in sealed plastic bags for 24 h to maintain humidity. Upon full maturity of siliques, seeds were harvested, surfaced sterilised and germinated on MS media containing BASTA (10 mg/L) and ticarcillin (50 mg/L).

### Genotyping

Genomic DNA from ~50 mg of leaf was isolated by grinding the tissue in 0.5 mL extraction buffer (0.2 M Tris‐Cl pH 9.0, 0.4 M LiCl, 25 mM EDTA, 1% SDS). After centrifugation at 13000*g* for 2 min, 350 μL of the supernatant was added to 350 μL isopropanol and nucleic acids were recovered by centrifugation at 13000*g* for 10 min. The supernatant was poured off, and the pellet was dried for 10 min. Nucleic acid was resuspended in 500 μL TE buffer. Genotyping of the mutants was performed by PCR (GoTaq, Promega) using RP, LB4 and RP, LP primer combinations (Table S4) for *fanci‐1* and *fanci‐2*, and T‐DNA insertions confirmed by sequencing. Primers were designed using iSect (https://signal.salk.edu/tdnaprimers.2.html).

### Transient expression in protoplasts and microscopy

Protoplasts were prepared from the leaf tissues of Arabidopsis as described previously (Wu et al., 2009). Leaves with the lower epidermis removed using magic tape were incubated for 1 h in digestion buffer (0.4 M mannitol, 10 mM CaCl_2_, 20 mM KCl, 20 mM 2‐(N‐morpholino)ethanesulfonic acid (MES) pH 5.7) containing 1% cellulose and 0.25% macerozyme with gentle shaking. After digestion, the protoplast suspension was transferred to 50 mL tubes avoiding the undigested leaf material and centrifuged at 100*g* for 3 min. The pellet was resuspended in W5 solution (154 mM NaCl, 125 mM CaCl_2_, 5 mM KCl, 5 mM glucose, 2 mM MES pH 5.7) and kept on ice for 30 min. The protoplasts were then pelleted by centrifugation at 100 g for 3 min and resuspended in 1.5 mL of MMg solution (0.4 M mannitol, 15 mM MgCl_2_, 4 mM MES pH 5.7). The number of protoplasts was determined using a hemocytometer. Approximately 10,000 protoplasts in 0.3 mL were transformed with 10 μg of each plasmid by addition of an equal volume of PEG buffer (40% (w/v) polyethylene glycol 4000, 0.1 M CaCl_2_, 0.2 M mannitol) for 5 min before dilution with 3 mL W5. Protoplasts were recovered by centrifugation at 100 g for 3 min and resuspended in 1.5 mL W5. Transformed protoplasts were incubated overnight at room temperature before imaging with a Zeiss Axiovert 880 inverted confocal microscope (www.zeiss.co.uk). pNOS:RFPnls was used as a transformation control. Quantification of cell death was performed using Zen software (Zeiss). Roots were stained with 10 mg/L propidium iodide immediately before imaging on the Zeiss Axiovert 880 and imaged with a x20 objective.

### DNA damage sensitivity assay

Sensitivity to mitomycin C was assayed by germination and growth of wild type and mutant lines in 3 mL ½ MS liquid media (Murashige and Skoog Basal Medium, Sigma‐Aldrich M551) supplemented with 2 mg/mL MMC in dimethyl formamide (DMF) to a final concentration of 6 μg/mL or DMF controls. Plants were grown in 6‐well plates at 22°C and 16 h light 8 h dark for 5 weeks (MMC treated) or 3 weeks (control) before determination of fresh weight. For UVC sensitivity, four seedlings from the wild type and each mutant were placed on square agar plates. The plates were then exposed to 0.5 to 2 kJ/cm^2^ of UVC light using UV crosslinker (FBUVXL‐1000 UV crosslinker, Fisher Scientific). Untreated plants were kept as a control. Both untreated and treated plants were incubated at 22°C and 16 h light 8 h dark and root length was measured every 2 days until day 6 post‐UV treatment. On the sixth day, the plants were photographed and the root length was measured. Hydroxyurea was used at 1 mM in half‐strength MS and seedlings grew vertically down the surface of the agar plate and root length quantified relative to those grown on control media. X‐ray treatment (150 Gy) was performed on 2d stratified seeds at a dose rate of ∼2 Gy/min using a 160 kV RS‐2000 X‐Ray Irradiator (Rad Source) and seeds then plated on half‐MS media and grown under standard conditions.

### Amplicon sequencing and bioinformatics analysis

Genomic DNA was extracted from pooled tissue from 20 to 30 primary transformed plants. The target locus was amplified using a pair of primers with 5' Illumina adapters (Tables S3 and S4) using 400 ng DNA and Q5 High‐Fidelity DNA Polymerase (NEB) using the following thermocycling conditions: 96°C for 2 min followed by 32 cycles of (98°C for 20s, 50°C for 20s and 72°C for 1 min) and a 5 min final extension at 72°C. Amplicons were purified using the PCR purification kit (Qiagen) according to the manufacturer's instructions. Paired‐end 250 bp sequencing was performed using Illumina technology (Genewiz). Sequence data were filtered using Trimmomatic to remove low‐quality reads and reads under 200 nucleotides in length (Bolger et al., 2014; Galaxy, 2024). Paired reads were used for the quantification of mutations at the target site using CRISPResso2 (Clement et al., 2019). Microhomology repair products were identified using https://www.rgenome.net/mich‐calculator/ (Bae et al., 2014). The number of modified reads for mutants was normalised to the number of modified reads for Col‐0 before repair product sequence analysis. Coexpression analysis and gene ontology enrichment were performed using https://www.michalopoulos.net/act/ (Zogopoulos et al., 2025). Analysis of post‐translational modification was performed using publicly available datasets accessed through the Athena website (athena.proteomics.wzw.tum.de).

### Statistical analyses

Data were analysed using R (R_Core_Team, 2020). Shapiro–Wilk tests (*P* > 0.05) were used to test for normality, and homogeneous variance was determined using Levene's test (*P* > 0.05). Analysis of variance (ANOVA) with post hoc Tukey tests was used for multiple comparisons. Kruskal–Wallis test with Bonferroni correction was used for analysis of data that was not normally distributed. Chi‐squared tests were used for comparisons between samples of read counts representing different DSB repair outcomes.

## Author Contributions

WMW, MKP and CEW supervised the experiments; AB, BC, VS, MRP, SFVN and WMW designed and performed experiments and analysed the data; AB, WMW, and CEW conceived the project, research plans and wrote the article with contributions from all the authors.

## Conflict of Interest

The authors declare no conflict of interest.

## Supporting information


**Figure S1.** *FANCI* expression in *fanci* mutants. Agarose gel electrophoresis of RT‐PCR products. Lane 1: Markers; Lanes 2–4: *ACTIN7* expression in *fanci‐1*, *fanci‐2* and Col‐0 detected using primers ACT7_F and ACT7_R; Lanes 5–7: *FANCI* expression in *fanci‐1*, *fanci‐2* and Col‐0. FANCI_F and FANCI_R primers span the sites corresponding to the T‐DNA insertion in both *fanci‐1* and *fanci‐2* in the *FANCI* cDNA.
**Figure S2.** Growth of *fanci* mutants. (a) Root length and (b) height of wild‐type and *FANCI* mutants. Data represent means ± SE. Different letters denote significantly different groups (*P* < 0.05, ANOVA with Tukey correction).
**Figure S3.** Genotoxin sensitivity of *fanci* mutant plants. (a) Rate of root growth of Col‐0, *fanci‐1*, and *fanci‐2* mutants. Roots were measured every 2 days until day 6 from control and UV‐treated plants (dose of 1600 mJ/cm^2^). (b) Mass of seedlings after 150Gy X‐ray treatment of 2d stratified seeds followed by 2 weeks growth on half‐MS media. (c) Root length of seedlings grown for 2 weeks on half MS with or without 1 mM hydroxyurea. Data represent means ± SE. Statistical significance was determined using ANOVA with Tukey correction, indicated by letters. *n* = 9–11. Data points represent individual plants.
**Figure S4.** Programmed cell death in the root apical meristem of *fanci‐2 mus81‐2* mutant plants. Laser scanning confocal microscopy of roots were stained with propidium iodide.
**Table S1.** Quantification of plant mass in response to MMC treatment.
**Table S2.** Quantification of cell death.
**Table S3.** Microhomology at the *IR10* locus.
**Table S4.** Microhomology at the *CAT* locus.
**Table S5.** Primer sequences.
**Table S6.** Guide RNA and the amplicon sequence.

## Data Availability

Sequence data are available as Supplementary Information. Other data are available on request. Genes analysed in this paper: *FANCI: AT5G49110, FANCD2: AT4G14970, MUS81: AT4G30870, BRCA1: AT4G21070*.
