# Supplementary material for: Arabidopsis thaliana FANCONI ANAEMIA I (FANCI) has roles in the repair of interstrand crosslinks and CRISPR‐Cas9 induced DNA double strand breaks
Source: Plant J. 2025 Oct 22;124(2):e70533. doi: 10.1111/tpj.70533 (PMC12541362; doi:10.1111/tpj.70533)
Supplement: Supplementary file 1 — Figure S1. FANCI expression in fanci mutants. Agarose gel electrophoresis of RT‐PCR products. Lane 1: Markers; Lanes 2–4: ACTIN7 expression in fanci‐1, fanci‐2 and Col‐0 detected using primers ACT7_F and ACT7_R; Lanes 5–7: FANCI expression in fanci‐1, fanci‐2 and Col‐0. FANCI_F and FANCI_R primers span the sites corresponding to the T‐DNA insertion in both fanci‐1 and fanci‐2 in the FANCI cDNA. Figure S2. Growth of fanci mutants. (a) Root length and (b) height of wild‐type and FANCI mutants. Data represent means ± SE. Different letters denote significantly different groups (P < 0.05, ANOVA with Tukey correction). Figure S3. Genotoxin sensitivity of fanci mutant plants. (a) Rate of root growth of Col‐0, fanci‐1, and fanci‐2 mutants. Roots were measured every 2 days until day 6 from control and UV‐treated plants (dose of 1600 mJ/cm2). (b) Mass of seedlings after 150Gy X‐ray treatment of 2d stratified seeds followed by 2 weeks growth on half‐MS media. (c) Root length of seedlings grown for 2 weeks on half MS with or without 1 mM hydroxyurea. Data represent means ± SE. Statistical significance was determined using ANOVA with Tukey correction, indicated by letters. n = 9–11. Data points represent individual plants. Figure S4. Programmed cell death in the root apical meristem of fanci‐2 mus81‐2 mutant plants. Laser scanning confocal microscopy of roots were stained with propidium iodide. Table S1. Quantification of plant mass in response to MMC treatment. Table S2. Quantification of cell death. Table S3. Microhomology at the IR10 locus. Table S4. Microhomology at the CAT locus. Table S5. Primer sequences. Table S6. Guide RNA and the amplicon sequence. [file TPJ-124-0-s001.zip › Supplementary Figures TPJ-00666-2025.docx]

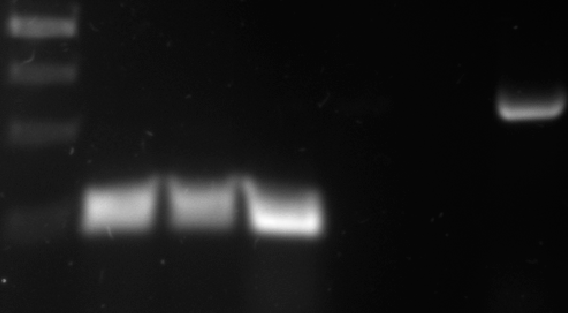


1 2 3 4 5 6 7

# Supplementary Figure 1.  *FANCI* expression in *fanci* mutants.

Agarose gel electrophoresis of RT-PCR products. Lane 1: Markers; Lanes 2-4: *ACTIN2* expression in *fanci-1*, *fanci-2* and Col-0 detected using primers ACT7_F and ACT7_R; Lanes 5-7: *FANCI* expression in *fanci-1*, *fanci-2* and Col-0. FANCI_F and FANCI_R primers span the insertion sites in both *fanci-1* and *fanci-2* in the *FANCI* cDNA.


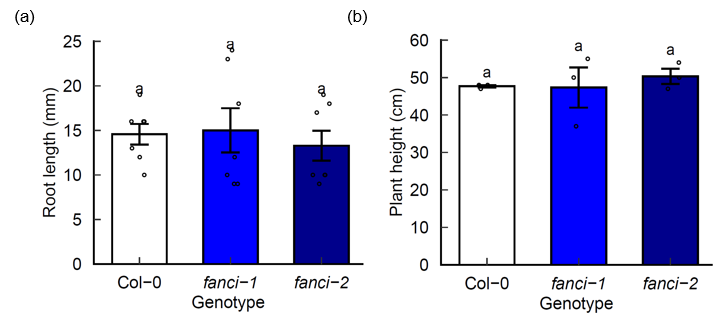


# Supplementary Figure 2.  Growth of *fanci* mutants.

(a) Root length and (b) height of wild-type and *FANCI* mutants. Data represent means ± SE. Different letters denote significantly different groups (P < 0.05, ANOVA with Tukey correction).

(a)


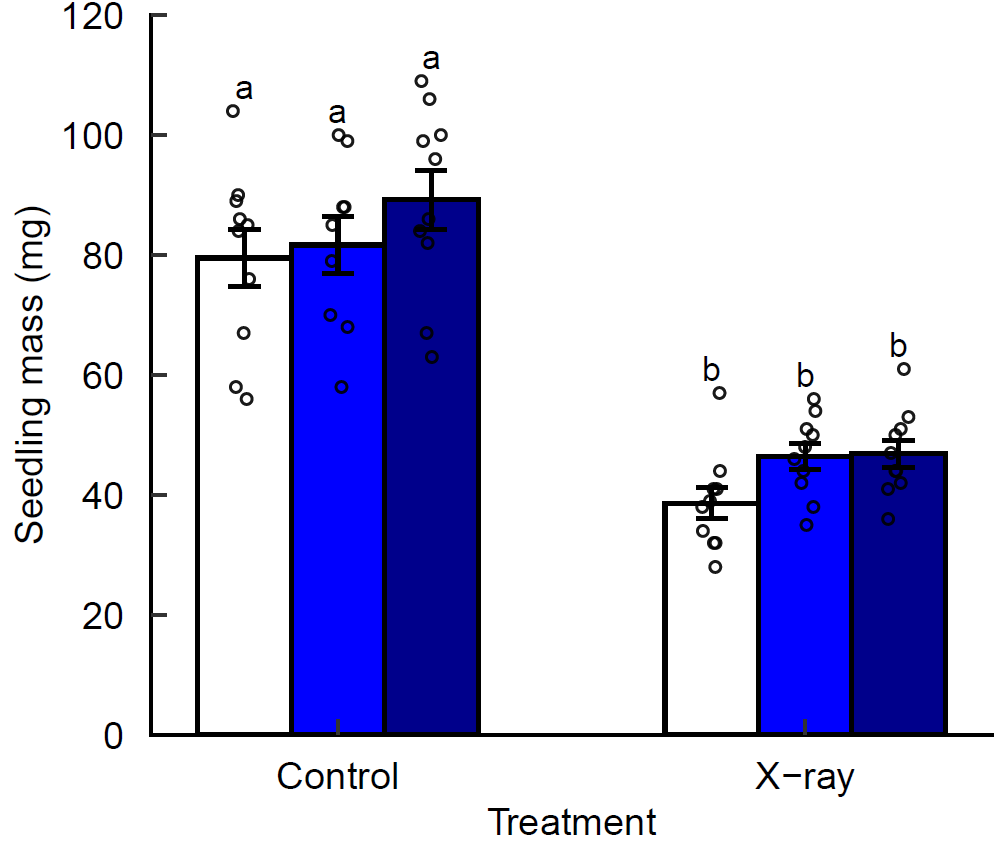

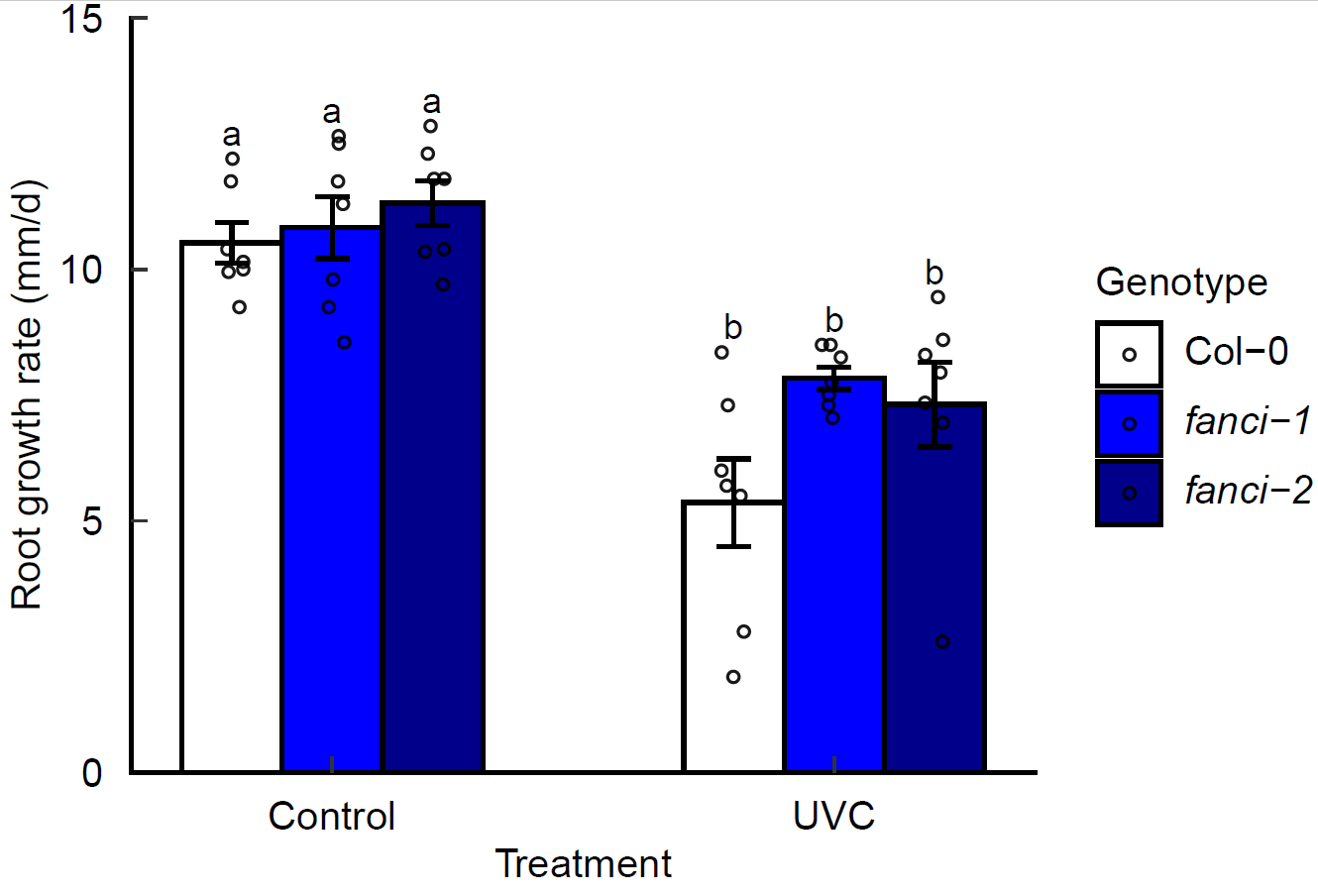


(b)

(c)


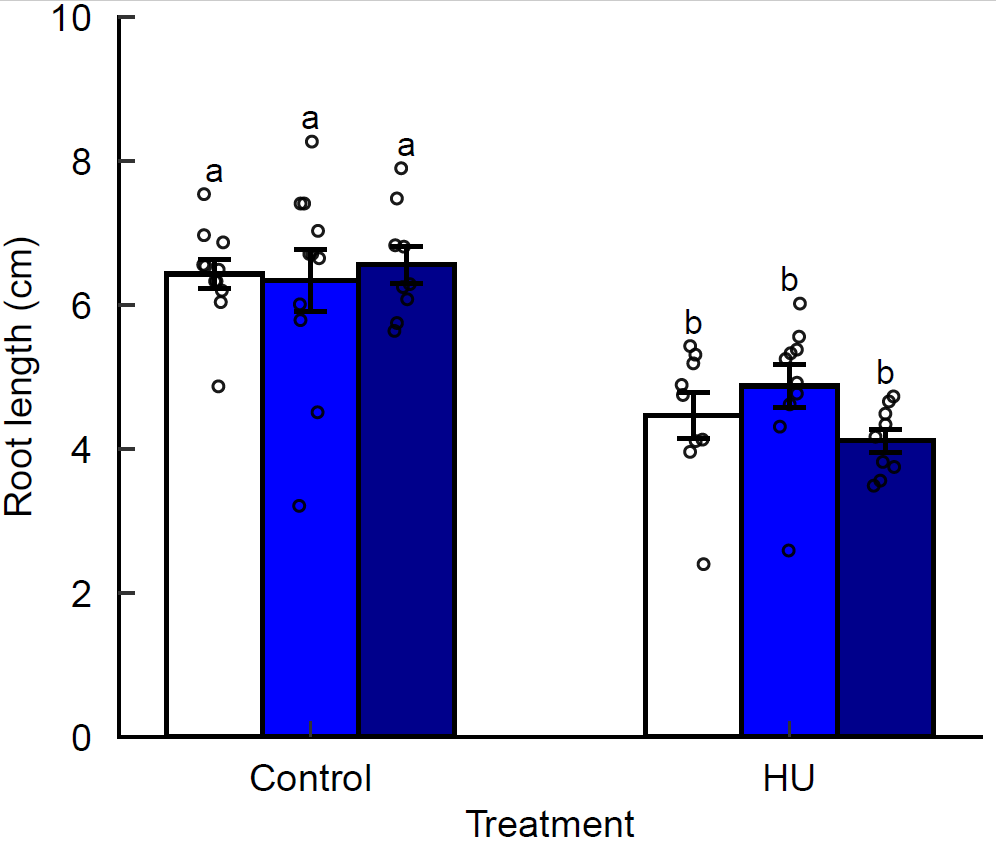


# Supplementary Figure 3. Genotoxin sensitivity of *fanci* mutant plants.

(a) Rate of root growth of Col-0, *fanci-1*, and *fanci-2* mutants. Roots were measured every two days until day 6 from control and UV-treated plants (dose of 1600 mJ/cm^2^). (b) Mass of seedlings after 150Gy X-ray treatment of 2d stratified seeds followed by 2 weeks growth on half-MS media. (c) Root length of seedlings grown for 2 weeks on half MS with or without 1mM hydroxyurea. Data represent means ± SE. Statistical significance was determined using ANOVA with Tukey correction, indicated by letters. n=9-11. Data points represent individual plants.


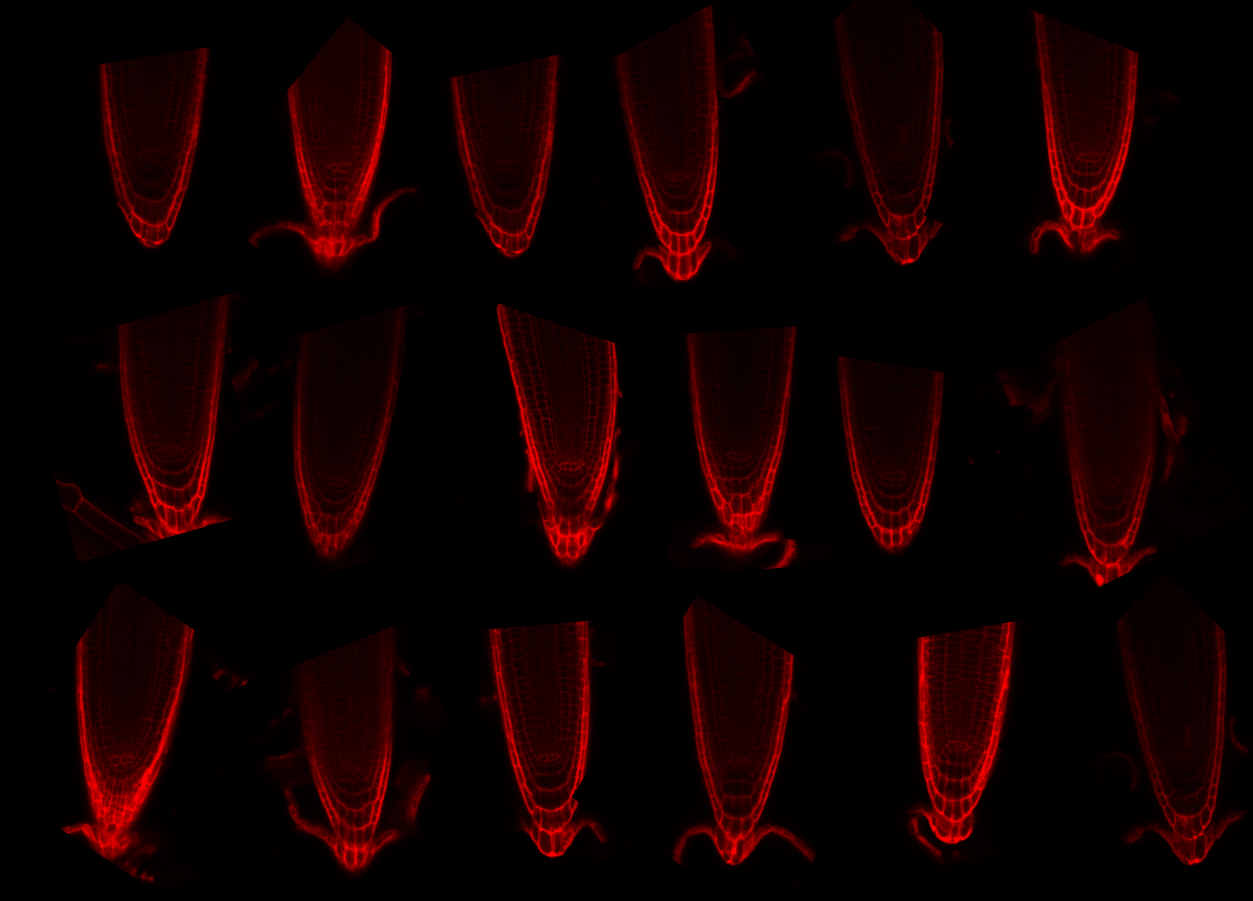


**Col-0**


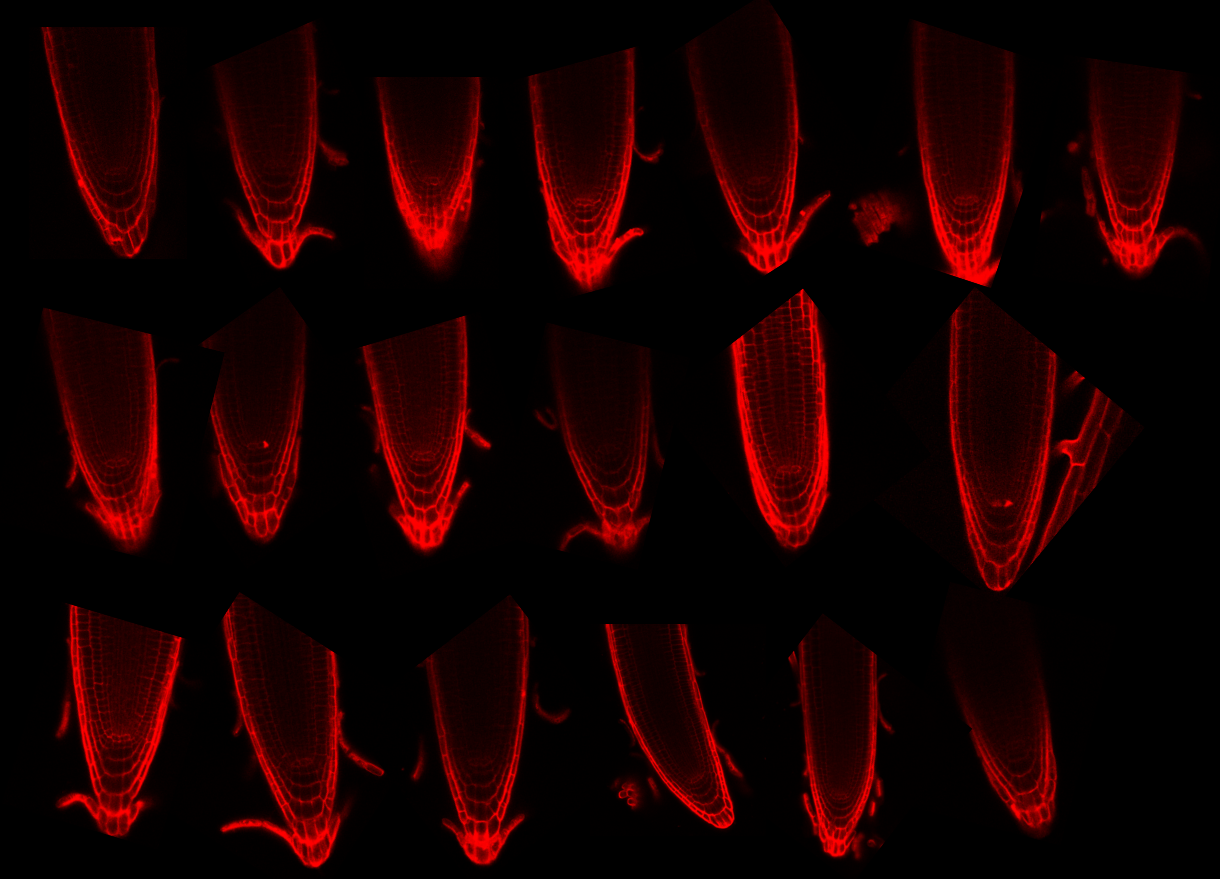


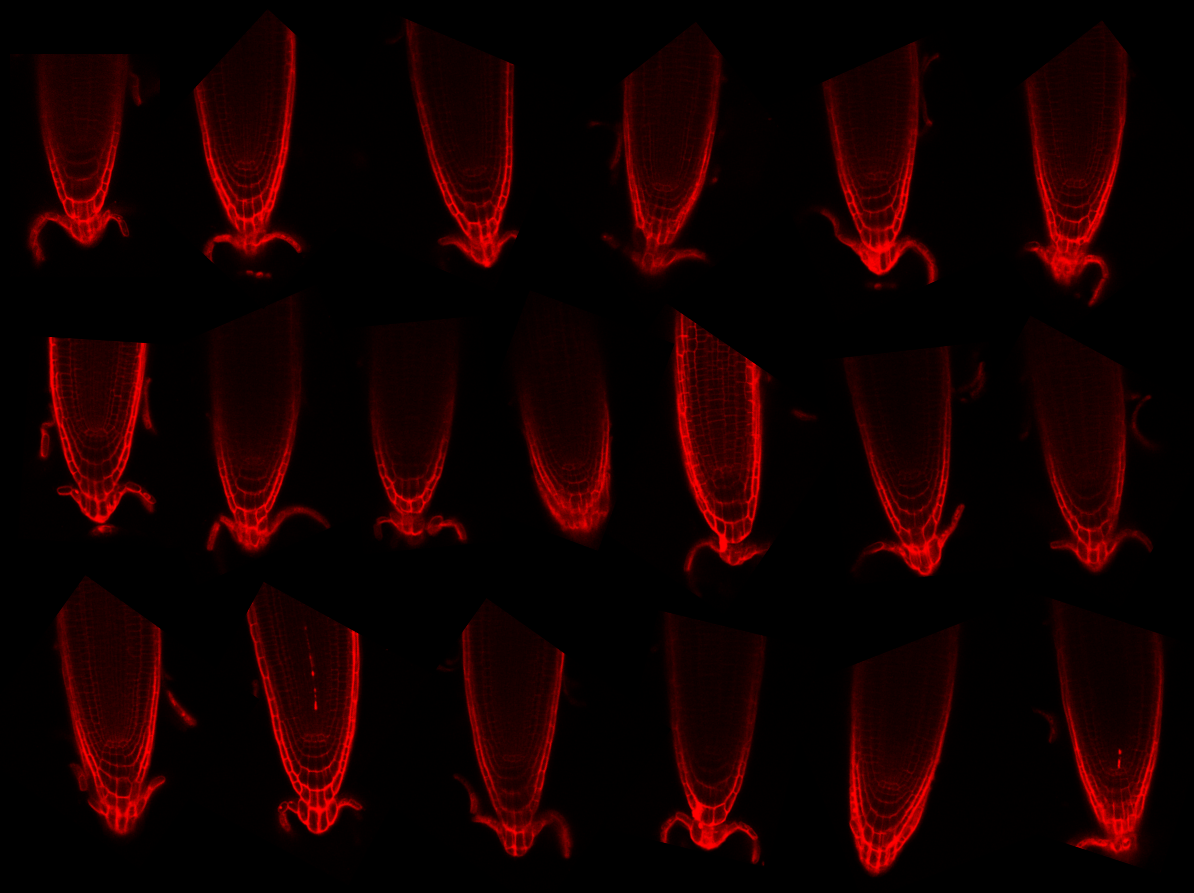


***fanci-2***

***fanci-1***


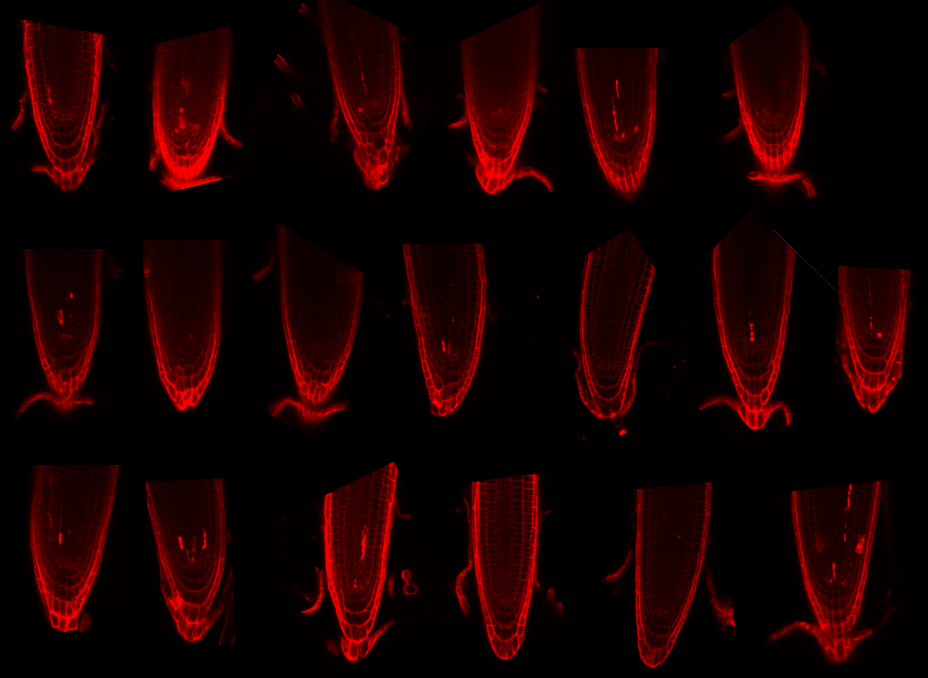


***mus81-2***

#
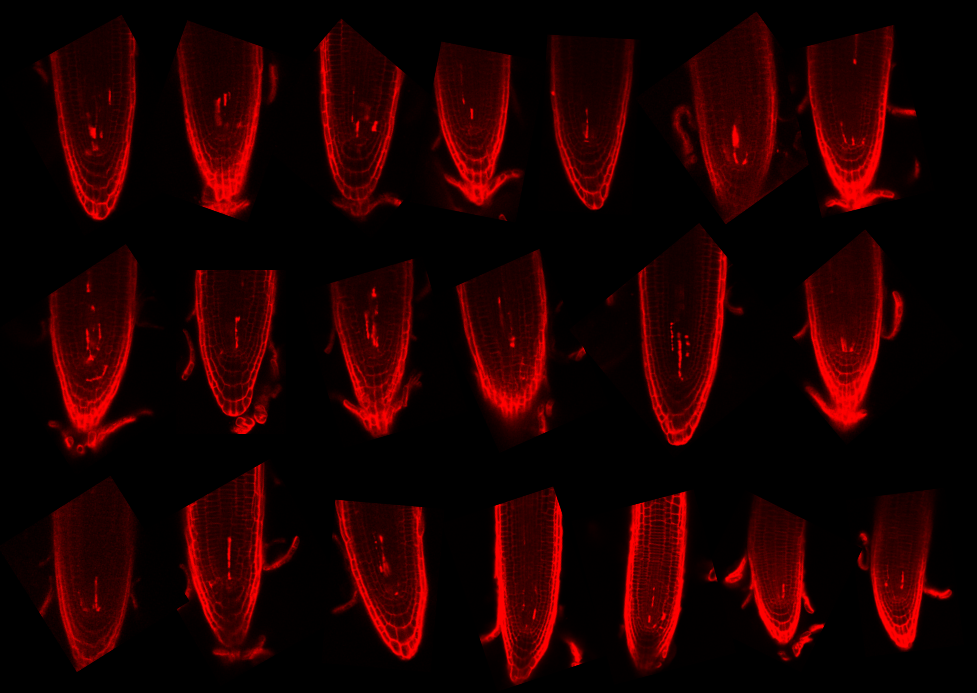


***fanci-1 mus81-2***

#

#
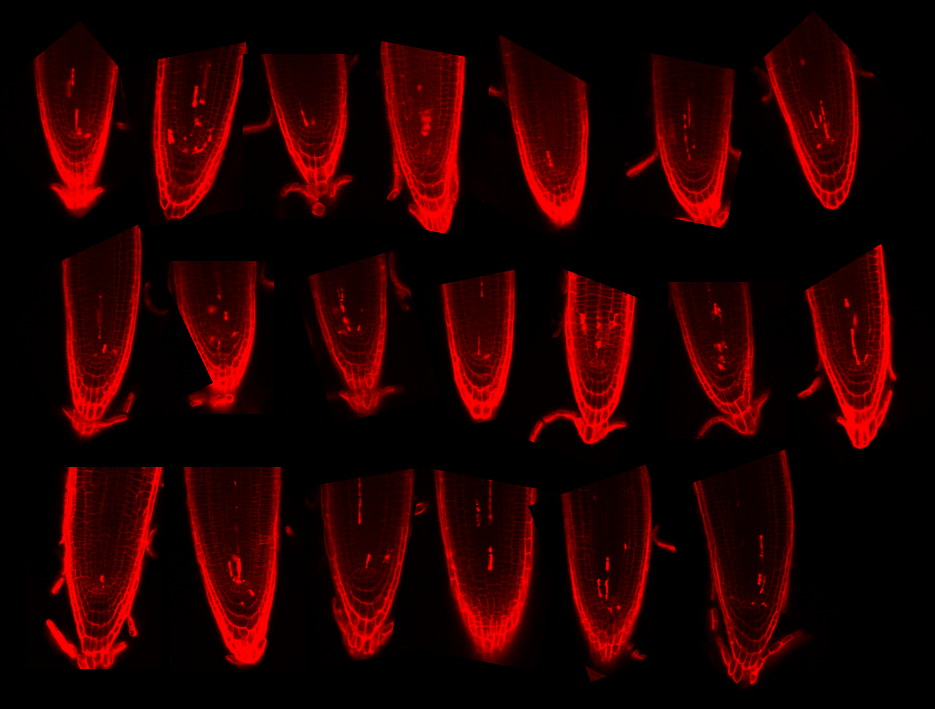


***fanci-2 mus81-2***

# Supplementary Figure 4. Programmed cell death in the root apical meristem of *fanci-2 mus81-2* mutant plants.

Laser scanning confocal microscopy of roots were stained with propidium iodide.

# Supplementary Table 1: Mass of MMC treated plants and controls (Data file)

# Supplementary Table 2: Quantification of cell death (Data file)

# Supplementary Table 3 Microhomology at the *IR10* locus

# Supplementary Table 4 Microhomology at the *CAT* locus

# Supplementary Table 5 Primer sequences

Primers

| Primers used for genotyping | *fanci-2* | LP :ATGGAACAAAGCGTTGTTGAC  RP : CTTTGCGCATTTCAAAGAAAG  LB4 :AACCACCATCAAACAGGATTTTCG |
| --- | --- | --- |
|  | *fanci-1* | LP AGTCCAACACATGTCCTCCAC  RP TGAGTTTGGTGATTCGAAAGG  LB4: AACCACCATCAAACAGGATTTTCG |
|  | *brca1-3* | LP TAATTGTGCCAGAAGTCCCAC  RP ATGAAACCTTCATCCCAAACC  LB4: AACCACCATCAAACAGGATTTTCG |
| Guide RNA primer | IR10 | IR10 F ATTG CAATCTCGTCCAGGATCCTA  IR10 R AAAC TAGGATCCTGGACGAGATTG |
|  | CAT | CAT3_F ATTG GACCATGGATCCTGCAGATG  CAT3_R AAAC CATCTGCAGGATCCATGGTC |
| RT-PCR primers | ACTIN7 | ACT7_F GTTCTACTCACAGAGGCACCTC  ACT7_R CCGTTCTGCGGTAGTGGTGAAC |
|  | FANCI | FANCI_F GTCGCGGTTCTGTTTTCCGTTG  FANCI_R TGAGCGGGATAACAGCAGAA |
| Sequencing primer | ILM IR10 F | ACACTCTTTCCCTACACGACGCTCTTCCGATCT CAACTTTTGGGTTCCGGGTC |
|  | ILM IR10 R | GACTGGAGTTCAGACGTGTGCTCTTCCGATCT GCCCATTGAAAATCCCTCGA |
|  | ILM CAT R | GACTGGAGTTCAGACGTGTGCTCTTCCGATCT TCCAAGGCGATGTCTCTGAG |
|  | ILM CAT F | ACACTCTTTCCCTACACGACGCTCTTCCGATCT ACTGATGAAGAGGCCAAGGT |
|  | ILM CAT F2 | ACACTCTTTCCCTACACGACGCTCTTCCGATCT CCACACCTACACTCTTATTGCC |
|  | ILM CAT R2 | GACTGGAGTTCAGACGTGTGCTCTTCCGATCT CTGTTCAGAACCAAGCGACC |

# Supplementary Table 6 Amplicon sequences

Guide RNA and the amplicon sequence used

| Site | sgRNA (PAM in red font) | Amplicon sequence |
| --- | --- | --- |
| CAT | GACCATGGATCCTGCAGATGAGG | ACTGATGAAGAGGCCAAGGTTGTTGGAGGAGCCAATCACAGCCACGCCACTAAGGATCTCCACGATGCCATTGCATCTGGCAACTACCCCGAGTGGAAACTTTTCATCCA**GACCATGGATCCTGCAGATG**AGGATAAGTTTGACTTTGACCCACTTGATGTGACCAAGATCTGGCCTGAGGATATTTTGCCTCTGCAACCGGTTGGTCGCTTGGTTCTGAACAGGACCATTGACAATTTCTTCAATGAAACTGAGCAGCTTGCGTTCAACCCGGGTCTTGTGGTTCCTGGAATCTACTACTCAGACGACAAGCTGCTCCAGTGTAGGATCTTTGCTTATGGTGACACTCAGAGACATCGCCTTGGA |
| CCX | CAATCTCGTCCAGGATCCTAGGG | CAACTTTTGGGTTCCGGGTCAAATAAACCCACCCAATTCAATTAGGATACTAATTAAGCAAGTTAAGATTCCGACACAAGTGATTCTCACCGATCGATGTTTCGTACGGAGAAAACGGTTTTCTGATCAGAGATCACGTCGATTCAAATCGGTGAGCATCTGAAAACTTTGATTCAATCTCGTCCAGGAT**CCTAGGGTTT**CA**AAACCCTAGG**TCTCTCTCCCGATTTGTGGTTTTGCAGCTAAATTACGCTATGATCTGTGGGGGGTAGTTGTTGTTGATAGGATTCAAGTTTTCAGATTTCGAAATGAGAGCTGTGAATTTTATGTATAGCTCGAATAATCCCAAATTTCGAGGGATTTTCAATGGGC |
| CAT  (used for fancd2) | GACCATGGATCCTGCAGATGAGG | CCACACCTACACTCTTATTGCCAAATCTGGAAAAGTTCTCTTTGTGAAGTTCCACTGGAAACCAACTTGTGGGATCAAGAATCTGACTGATGAAGAGGCCAAGGTTGTTGGAGGAGCCAATCACAGCCACGCCACTAAGGATCTCCACGATGCCATTGCATCTGGCAACTACCCCGAGTGGAAACTTTTCATCCA**GACCATGGATCCTGCAGATG**AGGATAAGTTTGACTTTGACCCACTTGATGTGACCAAGATCTGGCCTGAGGATATTTTGCCTCTGCAACCGGTTGGTCGCTTGGTTCTGAACAG |
